# Association of a New Germline Variant in the *MUTYH* DNA Glycosylase Gene with Colorectal Adenoma Transformation into Malignancy

**DOI:** 10.29252/ibj.23.6.412

**Published:** 2019-11

**Authors:** Amjad Mahasneh, Fawaz N. Al-Shaheri, Mohammed N. BaniHani

**Affiliations:** 1 Dept. of Applied Biological Sciences,Jordan University of Science and Technology, Irbid 22110, Jordan; 2 Dept. of Medical Laboratory Sciences, Jordan University of Science and Technology, Irbid 22110, Jordan; 3 Dept. of General Surgery and Urology, Jordan University of Science and Technology, Irbid 22110, Jordan

**Keywords:** Colorectal adenoma, Germline mutations, *MUTYH* gene

## Abstract

**Background:**

*MUTYH* DNA glycosylase germline mutations are linked to the recessive inheritance of multiple adenoma. Studies have revealed that germline mutations in this gene are ethnicity related. This study aimed to identify the germline mutations in *MUTYH* gene and determine their prevalence among Jordanian patients with colorectal adenoma.

**Methods:**

In this study, 150 colorectal adenoma patients and 150 cancer-free individuals with no previous history of polyps were recruited. Sanger DNA sequencing of the *MUTYH* gene (accession number NG_008189.1) was carried out using 3130xL Genetic Analyzer. Sequencing results were analyzed by ChromasPro, and mutational effects were predicted by online bioinformatics tools.

**Results:**

Two novel variants, g.87C>T and c.1264G>C, were identified. g.87C>T was also found in 60 (40%) patients and 10 (6.7%) controls. However, c.1264G>C was detected in 90 (60%) patients and 7 (4.7%) controls. Thus, a significant association was observed between these two variants and colorectal adenoma (*p* value for both variants was <0.0001). Moreover, the newly identified germline variant, c.1264G>C, was found to be significantly associated with colorectal adenoma transformation into malignancy *(p* < 0.0001).

**Conclusion:**

The data showed high prevalence of two germline mutations in* MUTYH *gene among Jordanians with colorectal adenoma, which may make them as potential early biomarkers for diagnosis of colorectal adenoma.

## INTRODUCTION

Colorectal adenoma pathogenesis is a multistep process and can induce neoplastic transformation of normal cryptic epithelial cells into neoplasia^[^^1^^]^. This stepwise transformation is due to multiple genetic and environmental factors, which disrupt the epithelial cells homeostasis by diminishing cellular apoptosis, persisting DNA replication, and losing the ability for differentiation and maturation^[^^2^^-^^4^^]^. Colorectal adenoma can be classified into tubular, villous, and tubuvillous adenoma^[^^5^^]^. The age, gender, smoking, diet, and obesity are environmental factors that contribute to colorectal neoplasia^[^^6^^-^^8^^]^. Genetic mechanisms such as chromosomal instability, CpG island hypermethylation, and microsatellite instability assist in colorectal neoplastic transformation. Microsatellite instability results from defect in DNA mismatch repair system that includes *PCNA*,* MLH1*,* MSH6*,* XRCC1*, and *MUTYH *genes^[^^9^^-^^11^^]^.

*MUTYH* comprises 16 exons encoding 535 amino acids protein and is mapped to the chromosome 1(1p32.1-p34.3)^[^^12^^]^. The MUTYH glycosylase, a base excision repair enzyme^[^^13^^]^, detects and repairs DNA damage including those generated by normal metabolic reactions such as alkylation, deamination, or oxidation^[^^14^^]^. The exposure of reactive oxygen species (produced during aerobic metabolism) to some chemicals/radiation influence the DNA integrity^[^^15^^]^. As a consequence, 7,8-dihydroxy-8-oxoguanine (8-oxo-G) often pairs with adenine (A), resulting in transversion of guanine:cytosine into thymidine:adenine (G:C> T:A) after two replication rounds of DNA^[^^16^^]^. The 8-oxo-G:A base-pair detection is initiated by MUTYH glycosylase^[^^14^^]^. 

*MUTYH* germline mutations have been linked to multiple colorectal adenomas inheritance in Caucasian populations^[^^17^^]^. Therefore, their identification greatly enhances our understanding of the colorectal cancer (CRC) potential causes and attributes in implementation of screening and management measures. Importantly, the early detection of CRC can be effective by establishing panels of genetic biomarkers. There are many genes and proteins currently used in the clinical settings in USA^[^^18^^]^, but they differ significantly in their predictive and prognostic values among populations^[^^19^^]^. Supposing that Jordanian patients with colorectal adenoma have a novel germline mutation(s) in *MUTYH* gene, this study aimed to identify these mutations among those patients and to determine their prevalence in relation to their ethnic background.

## MATERIALS AND METHODS

**Study subjects**

Colorectal adenoma patients (n = 150) were recruited from King Abdulla University Hospital (Ramtha, Jordan) during the period between January 2016 and February 2017. To select these patients, the biopsy samples from 400 subjects who visited the Endoscopy Unit at King Abdullah University Hospital were examined by a pathologist. Subjects (n = 150) who were confirmed pathologically to have tubular adenoma, villous adenoma, or tubulovillious adenoma were investigated in the study. Inclusion criteria include the number of polyps and the patient's previous treatment status. Patients with more than 10 polyps and without any surgical removal of polyps were enrolled in this study. However, those with a previous history of irritable bowel syndrome or Crohn’s disease were excluded. Clinical data were collected from patient's history files. The study was approved in advance by the Institution Review Board at King Abdullah University Hospital and Jordan University of Science and Technology (Ramtha, Jordan). Informed written consents were obtained from all participants. Besides, 150 cancer-free individuals with no previous family history of polyps were recruited as controls. A structured questionnaire interview was established to collect data on the characteristics of the study participants.

**Sample collection and handling**

One blood sample was collected from each participant. Five milliliters of whole blood was collected in EDTA tubes. Samples were transported on ice to the DNA extraction laboratory at Princess Haya Biotechnology Center (Ramtha, Jordan) and either processed immediately or stored at 4 C and extracted the next day. 

**Genomic** **DNA extraction**

DNA was extracted from whole blood samples using Qiagen® genomic DNA purification kit (Qiagen, USA) according to the manufacturer’s instructions. The concentration of DNA was determined using Nano Drop 2000 (Thermo Scientific, USA). To check the quality of extracted DNAs, two microliters from each extracted DNA sample was subjected to agarose gel electrophoresis. Extracted DNA samples were stored at -80 C.

**Polymerase chain reaction** **(PCR) procedure**

**Fig. 1:**
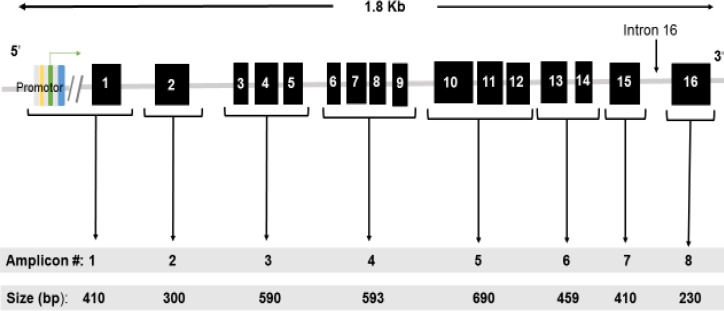
Schematic representation of the designed *MUTYH* amplicons.* MUTYH *5′untranslated region, 16 exons, and exons-intron boundaries were amplified in eight amplicons

All exons of *MUTYH *gene were amplified using PCR. The primers were designed using Primer3 Plus (http://www.bioinformatics.nl/cgi-bin/primer3plus/ primer3plus.cgi/). Eight amplicons were designed comprising the whole coding regions of *MUTYH* gene as well as the 5’ untranslated region and exon-intron boundaries (Fig. 1). Amplification of the target sequences was performed using conventional PCR (Veriti^TM ^Thermal Cycler from Applied Bioscience). PCR was carried out in a reaction volume of 25 L containing 12.5 L commercial 2 Master Mix (2.5 U* Taq* of DNA polymerase, 3 mM of MgCl_2_, and 0.5 mM of dNTPs), and buffer (100 mM of KCl, 20 mM of Tris HCl [pH8.3]), as well as 2L of each forward and reverse primer (10µM), 6.5 µl of nuclease-free water, and 2-3 µl of sample DNA. The list of primers and sequences for each amplicon are shown in Table 1. 

**Gel electrophoresis**

Five microliters from each PCR product was loaded on 2% agarose gel. The electrophoresis was run at 140 volts for 40 minutes. The running Tris/Borate/EDTA buffer was diluted to 1, and the bands were then detected under a UV light in Gel Doc^TM^ XR system (BioRad, USA) using Red Safe stain (iNtRON). Images were processed and analyzed by Quantity One 4.6.9 software.

**Cycler sequencing, cleaning and Sanger sequencing**

Purified PCR products were cycle-sequenced using Big Dye Terminator Ready Reaction (R-R) mix. The reaction components were 4 µL of sequencing buffer, 4 µL of nuclease-free water, 1 µL of R-R, 1 µL of forward or reverse primer, and 4 µL of purified DNA. The excess of Dye Deoxy^TM^ terminator, primer dimmers, and the other impurities were removed from DNA sequencing reaction using Qiagen cleaning kit. Sequencing of cleaned cycle-sequenced products was carried out on an ABI prism 3130xL Genetic Analyzer (Applied Biosystems, USA) in the Genomics Sequencing Laboratory at Princess Haya Biotechnology Center (PHBC). 

**Sequencing data analysis**

Sequence analysis was carried out using ChromasPro software (http://technelysium.com.au/wp/chromas/). 

**Bioinformatics and computational analysis of variants**

Several bioinformatics tools were used in this study. Primer3Plus online software (http://www. bioinformatics.nl/cgi-bin/primer3plus/primer3plus. cgi/) was used for primer design. MutationTastersoftware (http://www.mutationtaster.org/).PROVEAN (http://provean.jcvi.org/index.php) was applied for mutational effect prediction and PolyPhen-2 (http://genetics.bwh. harvard.edu/pph2/) for protein prediction analyses.

**Statistical analysis**

GraphPad Prism 6.0 was used for statistical analyses. In particular, *Chi*-square test was used. Statistical analyses with *p *value less than 0.05 were considered statistically significant.

## RESULTS

**Characteristics of the study participants**

The recruited Jordanian subjects with colorectal adenoma were 81(54%) males and 69 (46%) females, and the controls were 78 (52%) males and 72 (48%) females. There was no age difference between the patients and controls (*p* = 0.37, Table 2). Tubular adenoma was the most prevalent histopathogical group among 103 (68.6%) patients. Also, 28 subjects (18.6%) had villous adenoma, and 19 (12.7%) had tubuvillous adenoma. There was no significant difference in gender among the study subjects (*p* = 0.29; Table 3). Regarding the progression of colorectal adenoma into CRC, 107 (71.4%) subjects had progressed to CRC, while 43 (28.6%) had recovered after polypectomy. In addition, there was no significant association between CRC progression and gender (*p = *0.31). On the other hand, 86 (80.3%) patients, who progressed into CRC, continued to metastasis, compared to 21 (19.7%) who recovered after surgery and chemotherapy (*p* = 0.28).

**Table 1:** Primer sequences for *MUTYH* amplicons

**Amp** **licon**	**Reverse primer **	**Forward primer **	**Size (bp)**
1	ctttggatcacaacgctcaa	aggagacggaccgcaagt	410
2	gccaagagtaaacccgtgag	cttgggccacaacctagttc	300
3	catactgccacaggctgct	ggtctgacccatgacccttc	590
4	tggctatagaagtggcctaca	accccaacatcctaccagag	593
5	gcagtgttcccttcttttagg	cacgcccagtatccaggta	690
6	agggaatcggcagctgag	aaggaagtacaacaaagacaacaaa	459
7	acaaaagtactgggacatgaagtt	cctggagtggagaatgttca	239
8	gctctacagcattccaggcta	aatcacttgaggccagaatca	410

**Table 2:** General characteristics of the participants (n = 300)

**Variable**	**Control** **n (%)**	**Patient** **n (%)**	***p*** **value**
Gender			
Male	78 (52)	81 (54.0)	0.73^*^
Female	72 (48)	69 (46.0)	
Total	150	150	
Age groups (y)			
30-50	84 (56)	80 (53.3)	0.37
51-70	45 (30)	40 (26.7)	
<60	21 (14)	30 (20.0)	

^ *^Odds ratio: 0.922 and relative risk: 0.960

**Mutational analyses**

Interestingly, we identified five germline variants (g.87C>T, c.381A>C, c.724G>A, c.1264G>C, and c.1760C>T) in Jordanian subjects with colorectal adenoma (Table 4); two of which (g.87C>T and c.1760C>T) were novel. The c.1264G>C was the most common variant found in 90 patients (60%) with colorectal adenoma. Three variants (c.381A>C, c.724G>A, and c.1264G>C) were found to be disease predisposing, and two variants (g.87C>T and c.1760C>T) were considered as benign (Table 4). Based on Polyphen-2 protein prediction pipeline, c.724G>A and c.1264G>C were probably damaging to DNA glycosylase. However, c.381A>C and c.1760C>T were benign. These findings are consistent with those obtained from PROVEAN v1.1.3 software (http://provean.jcvi.org/seq_submit.php(, which indicates that c.724G>A and c.1264G>C are predicted to be deleterious to DNA glycosylase.

**Fig. 2:**
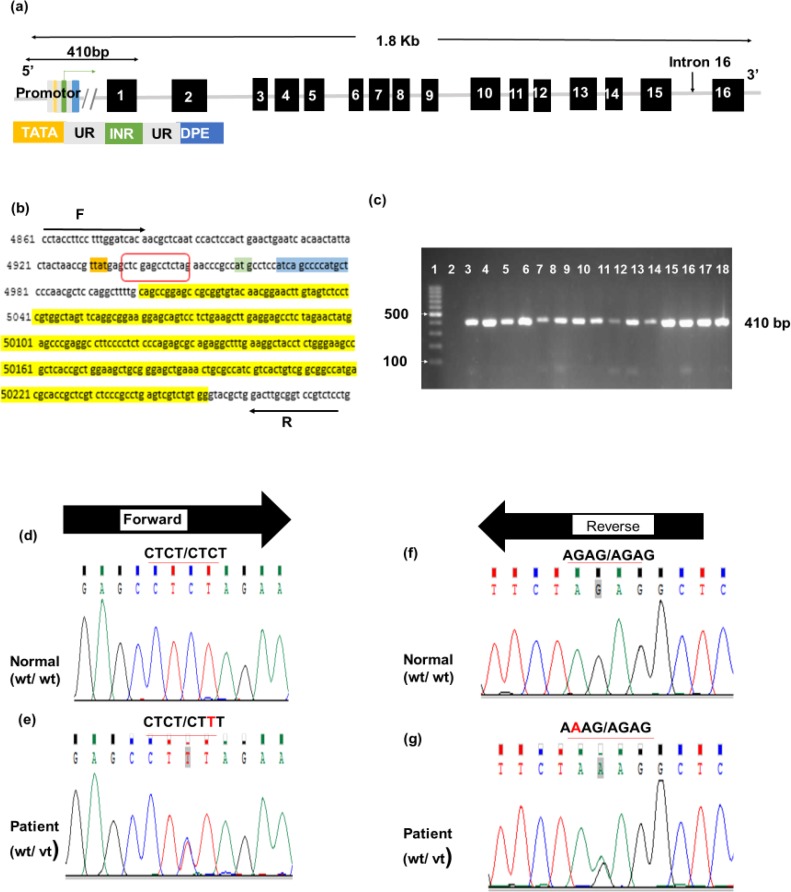
Mutational analysis of amplicon 1 of *MUTYH* gene. (a) Schematic representation of *MUTYH* gene showing that amplicon 1 contains 5’ untranslated region, exon 1, and the intronic boundaries. (b) The reference sequence of amplicon 1 obtained from Gene Bank (NG_008189.1) and the highlighted sequences are orange for TATA box, green for initiator, blue for downstream promotor elements (DPE), and yellow for exon 1. The red box contains the sequence in which g.87C>T variant was identified. The PCR was run using the forward and reverse primers (sequences above and below the block arrows). The expected PCR product was 410 bp. (c) 2% agarose gel electrophoresis of amplicon 1. The amplicon was successfully amplified and 410 bp bands were visualized using ethidium bromide staining. The size was compared to 100 bp ladder (lane 1), and the negative control (lane 2) was included. Lanes from 3-12 and from 13-18 are representative of patients and controls, respectively. (d) Partial chromatogram for forward primer sequencing of amplicon 1 from normal control, showing wild-type sequence (wt/wt). (e) Partial chromatogram for forward primer sequencing from patients illustrating heterozygous, g.87C>T, variant (wt/vt). (f) Partial chromatogram for reverse primer sequencing from normal control indicates wild type (wt/wt). (g) Partial Chromatogram for reverse sequencing from patients, showing heterozygous g.87C>T variant (wt/vt)

The heterozygous variant g.87C>T was identified in amplicon 1 in 60 subjects with colorectal adenoma and in 11 controls. Searching the ExAc and 1000 genome databases showed that g.87C>T was a novel variant found in this study. The variant was mapped to the 5′ untranslated region (5′UTR) of *MUTYH* gene (Fig. 2) and was named according to Human Genome Variation Society guidelines. Table 5 shows that g.87C>T is significantly associated with colorectal adenoma in comparison to the controls (*p* < 0.0001). Statistical analysis of allele frequencies of both groups showed that the wild-type allele (C) was more common in the control group (96.7%) than the patients group (80%). However, the mutant allele (T) was found in 20% among patients compared to 3.3% among the controls. These data are statistically significant with *p *< 0.0001.

**Table 3:** Clinical characteristics of the patients (n =150)

**Variable**	**Male** **n (%)**	**Female** **n (%)**	**Total** **n (%)**	***p*** **value**
Histopathology				
Tubular adenoma	60 (58.25)	43 (41.17)	103 (68.6)	0.29
Villous adenoma	13 (46.4)	15 (53.6)	28 (18.6)	
Tubuvillous adenoma	8 (42.10)	11 (57.9)	19 (12.7)	
Total	81	69	150	
CRC progression				
Yes	55 (51.5)	52 (48.5)	107 (71.4)	0.31^*^
No	26 (60.4)	17 (39.6)	43 (28.6)	
Total	81	69	150	
Metastasis				
Yes	42 (48.9)	44 (51.1)	86 (80.3)	
No	13 (61.9)	8 (38.1)	21 (19.7)	0.28^**^
Total	55	52	107	

^ *^Odds ratio: 0.692 and relative risk: 0.850;^**^odds ratio: 0.58 and relative risk: 0.788

**Table 4:** Genotype and allele frequencies of g.87C>T in *MUTYH* gene among study subjects (n =300).

**Genotypes and alleles**	**Control** **n (%)**	**Patient** **n (%)**	***p*** **value**
C/C	140 (93.3)	90 (60)	
C/T	10 (6.7)	60 (40)	
T/T	0 (0)	0 (0)	<0.0001
Total	150	150	
Allele C	290 (96.7)	240 (80)	<0.0001^*^
Allele T	10 (3.3)	60 (20)	
Total	300	300	
Familial	1 (10)	4 (6.7)	0.548^**^
Sporadic	9 (90)	56 (93.3)	
Total	10	60	

Odds ratio: 7.25and relative risk: 4.26; ^**^odds ratio: 0.642 and relative risk: 0.928

**Table 5:** Genotype and allele frequencies of germline mutation c.1264G>C in *MUTYH *gene in study subjects (n = 300)

**Genotypes and Alleles**	**Control** **n (%)**	**Patient** **n (%)**	***p*** **value**
G/G	143 (95.3)	60 (40)	<0.0001
G/C	7 (4.7)	5 (3.3)	
C/C	0 (0)	85(56.7)	
Total	150	150	
Allele G	293 (97.7)	125 (41.7)	<0.0001^*^
Allele C	7 (3.3)	175 (58.3)	
Total	300	300	
Familial	6 (85)	63 (70)	0.376^**^
Sporadic	1 (15)	27 (30)	
Total	7	90	

*Odds ratio: 58.6 and relative risk: 18.22; ^**^Odds ratio: 2.57 and relative risk: 2.43.

Amplicon 2 contains exon number 2 of *MUTYH* gene. It has been amplified to yield 300 bp PCR products. The PCR products were purified, and DNA cycle sequencing was carried out using the forward primer. However, no variant was found in this exon in the study subjects. Likewise, we carried out a PCR to amplify amplicon number 3, which contains three exons, 3, 4, and 5. This amplicon spans 590 bp as visualized by 2% gel electrophoresis. Analysis of amplicon 3 showed a reported single-nucleotide polymorphism called c.381A>C in 11 Jordanians with colorectal adenoma. c.381A>C was found to be disease predisposing with Bayes’s probability score of 50 (Table 7). Genotype frequency of both mutant (C) and wild type (A) among the study patients were 12 (8%) and 138 (92%), and their allele frequencies were 288 (96%) and 12 (4%), respectively among study patients (Table 6). Furthermore, amplicon 4 in* MUTYH* gene contains exons number 6, 7, 8, and 9. Overall, we identified a reported heterozygous variant, c.724G>A, in seven Jordanians with colorectal adenoma^[^^20^^]^. c.724G>A was found to be disease causing with a probability score of 61. It was also found probably damaging and deleterious for DNA glycosylase using PolyPhen-2 and PROVEAN pipelines, respectively (Table 7).

**Fig. 3:**
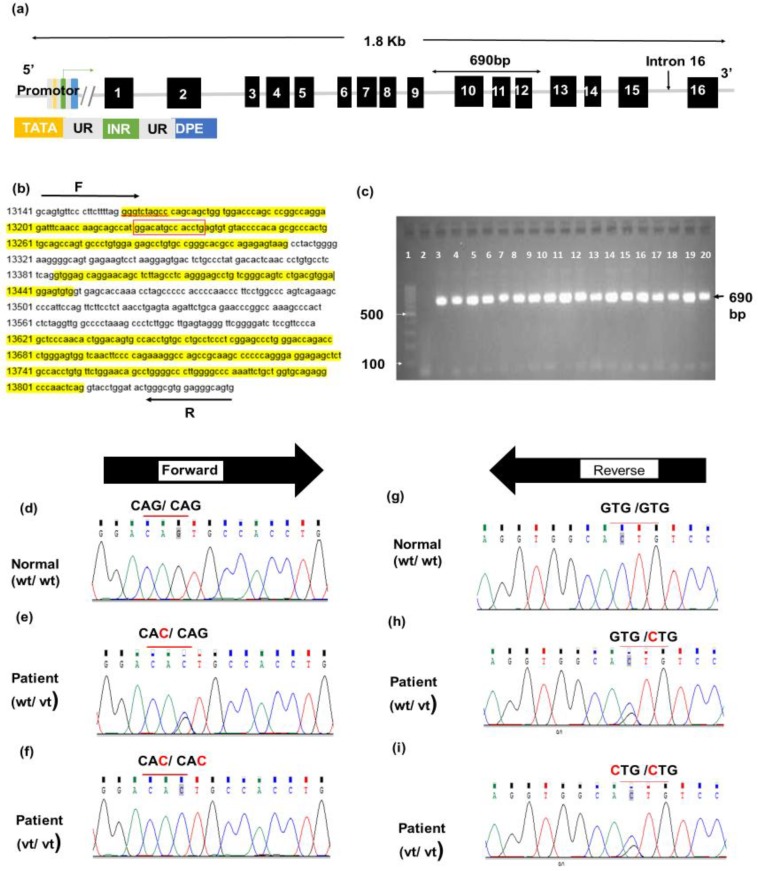
Mutational analysis of the amplicon 5 of *MUTYH *gene. (a) Schematic representation of *MUTYH*, showing that amplicon 5 contains three exons (10, 11, and 12) and the intronic boundaries. (b) The reference sequence of amplicon 5 obtained from Gene Bank (NG_008189.1) and the yellow highlighted sequences are for exons 10, 11, and 12. The PCR was run using the forward and reverse primers (sequences above and below the block arrows). The expected PCR product was 690 bp. The red box shows the position of c.1264G>C mutation. (c) 2% gel agarose gel electrophoresis for amplicon 5. The amplicon was successfully amplified, and 690 bp bands were visualized using ethidium bromide staining. The size was compared to 100 bp ladder (lane 1), and negative control (lane 2) was included. Lanes from 3-12 and 13-20 are representative patients and controls samples, respectively. (d) Partial chromatogram for forward primer sequencing of amplicon 5 from normal control showing wild-type sequence (wt/wt). (e) Partial chromatogram for forward primer sequencing from patients illustrating heterozygous, c.1264G>C, variant (wt/vt). (f) Partial chromatogram for forward primer sequencing from patient indicates homozygous c.1264 G>C mutation (vt/vt). (g) Partial chromatogram for reverse primer sequencing from normal control showing wild-type sequence (wt/wt). (h) Partial chromatogram for reverse primer sequencing from patient with heterozygous, c.1264 C>G, mutation (wt/vt). (i) Partial chromatogram for reverse primer sequence of amplicon 5 from patient with homozygous mutation, c.1264 C>G (vt/vt)

**Fig. 4:**
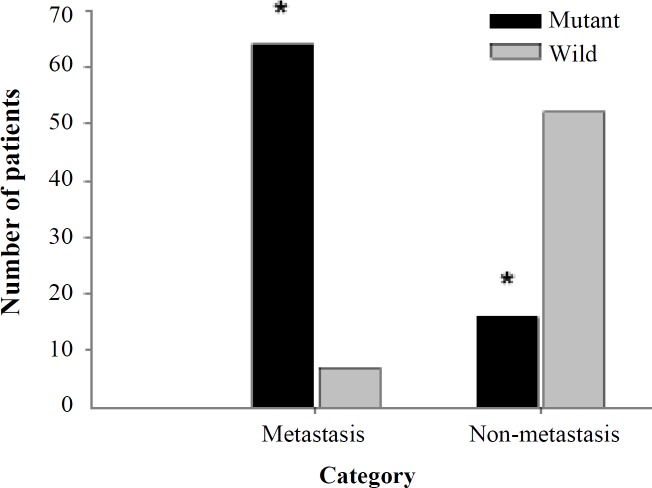
Association between c.1264G>C and colorectal adenoma malignant transformation. A strong association was found among the study subjects (*p* < 0.001). Of 90 subjects, who harbored the c.1264G>C germline mutation, 82 (91.2%) had progressed to colorectal cancer. In contrast, only 25 wild-type patients had progressed into CRC, comparable to 35 subjects who recovered after polypectomy

**Fig. 5:**
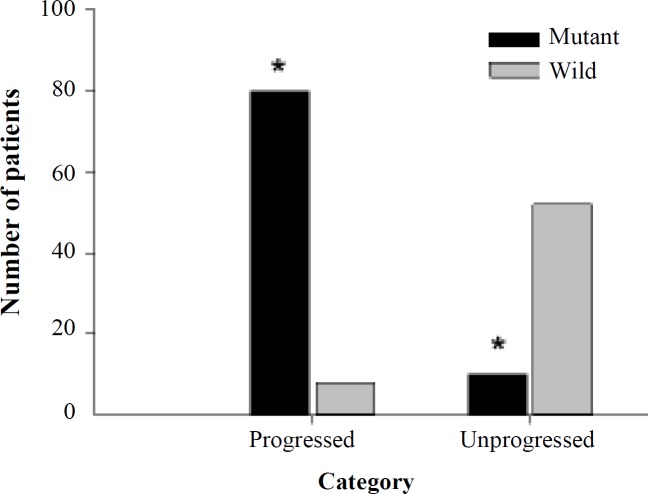
The association between c.1264G>C and CRC metastasis. A significant (*p* < 0.001) association was observed between c.1264G>C and CRC metastasis. Sixty four subjects included in this study with c.1264G>C germline mutation had CRC metastasis, comparable to 12 who had recovered after colectomy and chemotherapy

Amplicon 5 contains three exons, 10, 11, and 12. The size of PCR products is 690 bp (Fig. 3). Interestingly, the Sanger sequencing revealed a novel variant in 90 (60%) patients with colorectal adenoma from Jordan. As shown in Table 6), c.1264G>C was the highly prevalent variant allele detected in 90 (60%) patients under study. In particular, homozygous allele variant, C/C, was found in 85 (56.7%) patients, while G/C was found in 5 (3.3%) patients. Clearly, a significant association was observed between c.1264G>C variant and colorectal adenoma patients comparable to the controls (*p* < 0.0001). The mutation was mapped to position 1264 of the coding sequence of *MUTYH *(transcript usedNG_008189.1)*.* Besides, c.1264G>C was named according to Human Genome Variation Society guidelines. The mutation was predicted to be disease predisposing variant based on PROVEAN and PolyPhen-2. The normal codon, CAG of glutamine, at position 350 was altered into CAC that codes for histidine. PolyPhen-2 protein prediction analysis showed that the altered amino acid (p.Gln350His) would affect the protein structure and function. C.1264G>C was probably damaging and deleterious to DNA glycosylase based on data obtained from PolyPhen2 and PROVEAN, which scored 0.58 and -2.98, respectively (Table 7). The high prevalence of c.1264G>C in Jordanian patients with colorectal adenoma stressed the importance of investigating the association between this mutation and the progression of adenoma into carcinoma (Fig. 4). Interestingly, a significant association was observed between c.1264G>C mutation and malignant transformation of colorectal adenoma among patients (*p* < 0.001). This observation brings the necessity to evaluate the relationship between c.1264G>C and colorectal cancer metastasis. Hence, the disease pattern was followed, and the c.1264G>C was found to be significantly associated with CRC metastasis (Fig. 5).

**Table 6:** Genotype and allele frequency of germline variants found in Jordanian patients with colorectal adenoma (n =150)

**Amplicon** **number**	**Variant** **identified**	**Nucleotide** **(wt:vt)**	**Genotype frequency**			**Allele frequency** **(wt:vt) n (%)**
			**(wt/wt)** **n (%)**	**(wt/vt)** **n (%)**	**(vt/vt)** **n (%)**	
1	g.87C>T	C:T	90 (60)	60 (40)	0 (0)	C:240 (80) T:60 (20)
2	-	-	-			-
3	c. 381A>C	A:C	138 (92)	12 (8)	0 (0)	A:288 (96) C:12 (4)
4	c.724G>A	G:A	143 (95)	7 (5)	0 (0)	G:293 (97.6) A:7 (2.4)
5	c.1264G>C	G:C	60 (40)	5 (3.3)	85 (56.7)	G:125 (41.7) C:175 (58.3)
6	-	-				-
7	-	-				-
8	c.1760C>T	C:T	145 (96.7)	5 (3.3)	0 (0)	C:295 (98.3) T:5 (1.7)

Wt, wild type; vt, variant type

**Table 7:** Characteristics of germline mutations found in Jordanian patients with colorectal adenoma (n = 150)

**Amplicon** **Number**	**Variant**	**dbSNP ID** **(rs)**	**MutationTaster** **Prediction(score)**	**PolyPhen-2 prediction(score)**	**PROVEAN prediction(score)**
1	g.87C>T	Novel	Polymorphism (12)^*^	-	-
2	-	-	-	-	-
3	c. 381A>C	rs199929178	Disease predisposing (50)^*^	Benign (0.002)^**^	-
4	c.724G>A	rs786203212	Disease predisposing (61)^*^	Probably damaging (1.0)^**^	Deleterious (-2.863)^***^
5	c.1264G>C	Novel	Disease predisposing (81)^*^	Probably damaging (0.58)^**^	Deleterious (-2.98)^***^
6	-	-	-	-	-
7	-	-	-	-	-
8	c.1760C>T	rs140118273	Polymorphism (15)^*^	Benign (0.003)^**^	Neutral (-1.0)

^*^Bayes classifier score, which calculates the actual probability that certain alteration causes a disease. The range of result values is between 10 and 100. Therefore, the higher score, the more harmful alteration for human health. ^**^This score indicates the probability that certain mutation is damaging. Hence, variants scores between 0.0 and 0.15 are benign, and variants with scores between 0.15 and 0.40 are possibly damaging. However, variants that score between 0.51 and 1.0 are probably damaging. ^***^Delta alignment score of PROVEAN: if the score is below or equal to the threshold (-2.5), the alteration is predicted to have deleterious effect. However, if the variant has a score above -2.5, the variant is predicted to be neutral.

Exon 15 and its intronic boundaries (amplicon 7) were also amplified using the primers listed as 7 in the Table 1. PCR was successfully performed and resulted in amplification of 230-bp products. The products were purified and sequenced using 3130xL Genetic Analyzer (Applied Biosystem, USA). However, no mutation was found in this exon among the study subjects. 

Finally, exon 16 and its intronic boundaries (amplicon 8) were amplified using the listed primer 8 (Table 1). PCR successfully amplified 410-bp products. The products were purified and sequenced using 3130XL Genetic Analyzer. A reported variant, rs140118273, was successfully identified in Jordanians with colorectal adenoma. However, the variant was found to be neutral.

## DISCUSSION

In this study, five variants were found in Jordanian patients with colorectal adenoma, of which three (c.381A>C, c.724G>C, and c.1760 C>T) has previously been reported, and two (g.87C>T and c.1264G>C [Q350H]) were novel. These findings are in contrast to Caucasian populations in which Y165C and G382D are the most common mutations associated with multiple polyposis syndrome^[^^18^^]^. The Y165C and G382D mutations have been shown to impair the MUTYH repair activity and significantly contribute to CRC development^[^^19^^]^. The missense R245C and the splice site IVS10-2A>G variants have been identified in Japanese and Korean patients^[^^15^^,^^20^^]^. In another studies conducted in Korean and Japanese patients (n = 97), 7.2% were bi-allelic carriers for the germline mutations c.1-18G>T, A359V, and R170G in the *MUTYH* gene^[^^21^^,^^22^^]^. After screening the entire gene, Vandrovcová*v et al.*^[^^23^^] ^found no mutation in *MUTYH* in Singaporean population, though this may be a biased result due to the small sample size (n = 63). Furthermore, five unrelated Indians patients with colorectal adenoma were homozygous for the missense mutation E480X^[23]^. Other *MUTYH* variants have been found in Italian (A473D), Finnish (p.P391L), Swedish (p.G175E), and Portuguese (E383fsX45)1^[^^24^^-^^27^^]^. In a case-control study and meta-analysis, Win *et al.*^[^^26^^]^ have indicated an association between *MUTYH* gene mutations and the increased CRC risk. Another meta-analysis demonstrated the risk of *MUTYH* in monallelic and biallelic carriers^[28]^. *MUTYH* gene loss has been linked to colorectal carcinogenesis due to immunosuppression and altered immune response^[27]^. Grasso *et al.*^[^^28^^]^ have shown that *MUTYH *mediates the toxicity of DNA 6-thioguanine and UV radiation. MUTYH-OGG1 XRCC1-PARP1-MMP1 is a linear interacting susceptibility locus for CRC^[^^29^^]^.

In Arabian descent populations, four studies have been performed; one in Tunisia that showed high prevalence of c.1227-1228dup^[^^30^^]^, the second in Morocco that introduced three variants (c.494A>G, c.1145G>A, and c.1185_1186dup)^[^^31^^]^, the third in Saudi Arabia that revealed the presence of V22M, Y165C, H324Q, and G382D variants^[^^32^^]^, and the last in Egypt that found a significant association between G396D and Y179C mutations and colorectal carcinogenicity^[^^33^^]^. These results contradict with those obtained from a study involved 360 Arabic patients^[^^34^^]^. The best explanation for these discrepancies between data is that these studies only targeted either specific reported mutations, such as G396D and Y179C, or familial cases. However, in the present study,* MUTYH* gene was sequenced thoroughly for the patients with colorectal adenoma and the controls from the same ethnic group. It has recently been shown that *MUTYH* interacts with *Rad9-Rad1-Hus1* complex (9-1-1 complex) to coordinate the cell cycle checkpoint. The interdomain connector (IDC) of *MUTYH* (residues between 65 and 350) is critical for 9-1-1 complex and *MUTYH *interaction^[11]^. The significance of the interaction between* SpMyh1*I DC and 9-1-1 has been elucidated by Luncsford *et al.*^[11]^ who tested *in vivo *the biological effects of V315 and I261 mutations in the impairment of 9-1-1 complex-*MUTYH* interaction. Interestingly, they showed that *MUTYH* IDC is important for *MUTYH* DNA repair function and interaction with 9-1-1 complex. *MUTYH* IDC aspartate at the position number 350 interacts with histidine at the position number 18 of 9-1-1 complex^[^^35^^]^. Therefore, c.1264G>C missense variant found in Jordanian patients would probably affect *MUTYH*-9-1-1 complex interaction. This finding explains the strong association between c.1264 G>C and colorectal malignant transformation among mutant colorectal adenoma patients. However, this hypothesis requires further functional testing using cell-based cloning and cell culture manipulation. 

 In conclusion, germline mutations in *MUTYH* gene are highly prevalent among patients with colorectal adenoma from Jordan. In particular, c.1264 G>C is the most common variant associated with malignant transformation of colorectal adenoma into carcinoma. This variant has a potential to be an early biomarker for diagnosis of CRC in Jordan.
